# Interfacing a Cary 210 spectrophotometer to a Commodore PET 2001 microcomputer

**DOI:** 10.1155/S1463924686000081

**Published:** 1986

**Authors:** D. Migneault, R. Mattera, R. K. Forcé

**Affiliations:** University of Rhode Island Chemistry Department Kingston Rhode Island 02881 USA


Journal of Automatic Chemistry/*Journal of Clinical Laboratory Automation*, Volume 8, Number 1 (January–March 1986), pages 32–34

### Interfacing a Cary 210 spectrophotometer to a Commodore PET 2001 microcomputer

**D. Migneault, R. Mattera and R. K. Forcé**

*University of Rhode Island, Chemistry Department, Kingston, Rhode Island 02881, USA*

### Introduction

The use of microcomputers for instrument control and data acquisition has become an important aspect of the chemical laboratory. The availability of inexpensive microcomputers, the ease of interfacing with existing chemical instrumentation, and the availability of such high-level languages as BASIC, all make such applications attractive and feasible to implement. A considerable amount of well-built instrumentation without microprocessor control exists and will be with us for some time. Interfacing brings this instrumentation into the current age at a cost far below that of purchasing a new instrument.

The authors have been acquiring UV scans by means of a simple microcomputer interface for some time. Benefits include a greater precision than is possible by hand digitizing, more facile data handling of larger data-bases, and, consequently, the acquisition of a much higher point density in each scan. High point density is an important consideration in such statistical procedures as factor analysis. Once the data is acquired by the microcomputer it can be processed by the same processor or passed along by a modem or RS232 interface to another more powerful computer.

This paper reports a method of interfacing a Cary 210 spectrophotometer (Varian Associates, Inc., 611 Hansen Way, Palo Alto, California 94303, USA) to a PET 2001 microcomputer (Commodore Business Machines, 201 California Avenue, Palo Alto, California 94303, USA). The interface is accomplished through the memory expansion port of the PET and utilizes a MCS-6522 Versatile Interface Adapter (MOS Technology, Inc., Valley Forge Corporate Center, 950 R. Henhouse Road, Norristown, Pennsylvania 19401, USA). The interface chip manages absorbance data output from the Digital Interface Port (DIP) of the Cary, handshaking signals necessary for managing the information transfer, and control commands issued by the PET to the Cary. This interface design is similar to one described by Grenier[1], which used a 6502-based Apple II Plus microcomputer and a 6821 interface chip.

### Method

The MCS-6522 Versatile Interface Adapter is a very powerful interface chip and is well suited for this application. It consists of two eight-bit bidirectional ports, Ports A and Port B, as well as four control lines. One port is used to input data from the Cary and the other to output data to the Cary. Two control lines are used to manage the handshaking commands, as well as two lines of Port B. It also has on board a 16-bit timer which counts TTL pulses incoming on line 6 of Port B, as well as an interrupt flag register. Other features of this chip are not used in this application.

The 6522 is mounted on a single Vector 3677-2 circuit board (Vector Electronic Company, 12460 Gladstone Avenue, Sylmar, California 91342, USA), and is powered by an auxiliary +5 V power supply. Also mounted on the circuit board is a DM-7404 hex inverter (National Semiconductor Corporation, 2900 Semiconductor Drive, Santa Clara, California 95051, USA).

The connections between the PET and the 6522 are simple and straightforward (figure 1). The data bus is connected directly, as well as the reset, clock and read/write. IRQ is not connected in this application. The PET contains a 16-bit address bus. To operate the interface chip only five of the address lines are utilized. Turning on the chip is accomplished by utilizing only $\overline{SEL6}$ of the PET. $\overline{SEL6}$ selects the 4K byte page of memory with a base address of 24576. In our PET, an early 8K model which is equipped with a 16K expansion board (Skyles Electric Works, 231 E. South Whisman Road, Mountain View, California 94041, USA), nothing is located in this page except this interface chip. $\overline{SEL6}$ is connected directly to $\overline{CS2}$ and through the inverter to CS1. When a location in the 4K page is addressed CS1 is driven high and $\overline{CS2}$ driven low, simultaneously activating the chip.

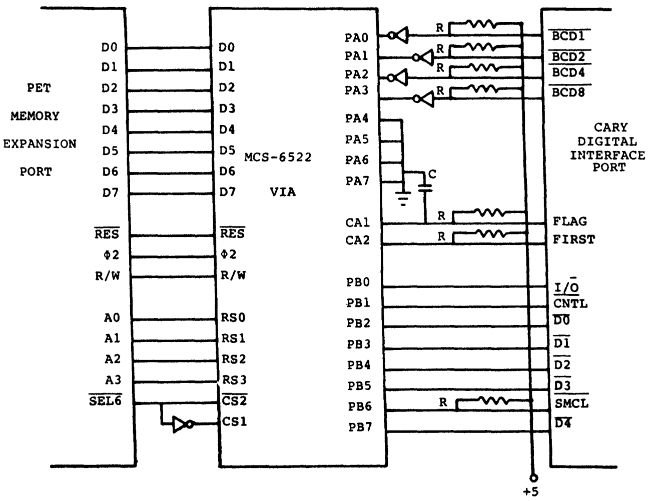

*Figure 1. Schematic diagram of the PET-Cary interface showing connections to the MCS-6522 Versatile Interface Adapter, R = 3.3 Kohm, C = 470 pF.*

The middle eight lines of the address bus are not connected. If more devices such as additional 6522s were to be placed in this page of memory, these lines could be utilized for the address of the chip by demultiplexing these eight lines to control CS1, leaving $\overline{\text{SEL6}}$ to control $\overline{\text{CS2}}$. The four least significant lines of the address bus, AØ-3, are used to select registers on the 6522. Any of the 16 registers on the 6522 can be selected by using a base address of 24832 plus the register address.

*Table 1. MCS-6522 versatile interface adapter*

| Register addresses | |
|---|---|
| *Base +* | *Register* |
| 0 | Output, Port B |
| 1 | Output, Port A |
| 2 | Direction, Port B |
| 3 | Direction, Port A |
| 4–7 | Timer 1 |
| 8–9 | Timer 2 |
| 10 | Shift register |
| 11 | Auxiliary control |
| 12 | Peripheral control |
| 13 | Interrupt flag |
| 14 | Interrupt enable |
| 15 | Output, Port A |

The interface between the 6522 and the DIP is made through the 25-pin RS232-type connector of the Cary. The absorbance data is input through Port A of the 6522. Each output line from the DIP has a device with an open-collector output which requires the receiving circuit to be pulled up through a 3 KΩ resistor to +5 V (figure 1). Because the logic is true negative in the Cary and true positive in the PET, the four BCD lines are inverted after being pulled up and before being input into Port A. The remaining four lines to the Port A data bus, PA4-7, are grounded on the circuit board to provide logic Ø.

Two sensing control lines, CA1 and CA2, are input to the 6522 from FLAG and FIRST on the Cary. PBØ and PB1 are the control lines from the 6522 to I/$\overline{\text{O}}$ and $\overline{\text{CNTL}}$ on the Cary. These four lines govern all handshaking to manage information transfer. The five-bit control commands to the Cary are output on Port B lines PB2-5,7 to the Cary. PB6 is a specially programmable line on the 6522 connected to timer 2. It counts incoming pulses and sets a flag in the interrupt flag register (IFR) on the 6522 when a preset number of pulses are counted. Since the Cary outputs 100 pulses per nanometer on $\overline{\text{SMCL}}$ when scanning, the wavelength counting pulses on this line with this timer serves to monitor the wavelength of the Cary.

To conduct a scan, the Cary is first manually base-lined. Then it is set to the initial wavelength. The initial wavelength, as well as the interval between readings and number of readings, are programmed into the PET. The PET issues the commands to the Cary to scan down, stop and take absorbance readings at the wavelengths desired. When the end of the scan is reached, the scan direction is reversed and the Cary reset to the initial wavelength. Data is presented on the screen, on the printer and optionally stored on disk with user-developed routines written in BASIC.

Two aspects of the system control deserve special discussion: first, the issuing of commands by the PET to control the scanning of the Cary from one wavelength to the next; and, second, the transfer of absorbance information from the Cary to the PET. First, to scan across any given wavelength region, the following occurs: the number of pulses to be counted for the scan is calculated, divided into two eight-bit bytes and 'poked' into the two registers of Timer 2. The timer is then programmed to count incoming pulses and to set a flag in the interrupt flag register (IFR) on the 6522 when the required number of pulses has been counted. The PET polls this IFR to watch for the setting of the appropriate flag, which indicates that the predetermined number of pulses has been counted. Lines 2–5 and 7 of Port B are programmed by the Port B data direction register as output. Command control data is output on these five lines of the Port B.

A command is issued to the Cary by the following series of events: I/$\overline{\text{O}}$ is held low, $\overline{\text{CNTL}}$ is driven high, and the data placed on PB2-5, 7 of the 6522. After a 20 ms delay, $\overline{\text{CNTL}}$ is driven low initiating the data transfer to the Cary. When the CARY accepts the control data, it responds by driving $\overline{\text{CNTL}}$ high again and the process is complete. The input and output timing diagrams are schematically shown in the Digital Interface Port Operator's Manual [2]. Initial operating conditions of the Cary are issued in BASIC. However, the scan down command, and the polling for the flag set in the IFR when the predetermined number of pulses are counted, are both done in an assembly language subroutine in order that no pulses are missed and the scan interval is accurately accomplished.

The second aspect of system control to be discussed is acquiring an absorbance reading. When the Cary is stopped, the PET inputs the absorbance data from the Cary. The data consists of 18 four-bit nybbles of BCD information, which are output by the Cary in less than 54 ms, a timing constraint imposed by the Cary. Because of this constraint this subroutine is also written in assembly language. The subroutine stores these 18 nybbles for later conversion in BASIC to decimal values.

To accomplish a data transfer from the Cary, Port A is programmed as an input and Port B as an output. I/$\overline{\text{O}}$ and $\overline{\text{CNTL}}$ are held high for a minimum of 20 ms, whereupon $\overline{\text{CNTL}}$ is driven low. The Cary responds by driving FLAG low, indicating data is valid, and the PET latches the data and drives $\overline{\text{CNTL}}$ high. The Cary indicates data is no longer valid by driving FLAG high. This process is repeated 18 times to complete a data transfer.

### Results

To demonstrate the use of this interface the vapor phase spectra of benzene was taken. The Cary was base-lined using two matched 1cm quartz cells and two drops of

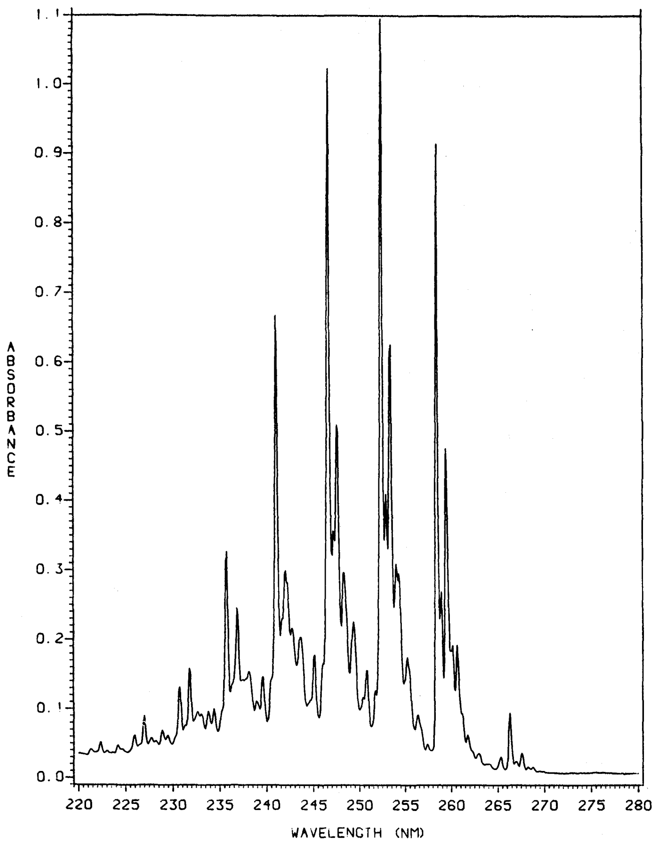

*Figure 2. Benzene vapor spectrum.*

reagent grade benzene were put in the bottom of the sample cell. After a sufficient period of time to allow for the benzene vapor to equilibrate, the scan was conducted with a 0 to 1 absorbance range and a bandwidth of 0.25 nm. A total of 601 data points were taken over the wavelength range of 280–220 nm and output to a floppy disk. The absorbance-wavelength data pairs were read by a second program and output to a Data General Eclipse S/130 minicomputer. The data file was converted into a program file in the word-processor and the data was submitted by remote job entry to the University of Rhode Island mainframe computer, a National Advanced Systems 7000 for graphic display (figure 2), by means of SAS Graphics Routines (SAS Institute, Inc. [3]). Typically, graphs are available 15 min after the scan is conducted.

Documented versions of the machine code are available from the authors, as well as copies of the BASIC programs which include this machine code.

### Acknowledgements

The authors wish to thank David Stout and Tim Wasco for their helpful discussions and comments with regards to the implementation of the MCS-6522. This work was supported in part by a grant from the University of Rhode Island Alumni Foundation.

### References

1. Grenier, J. In *Varian Instruments at Work, Number UV-4* (Varian Associates, Inc., 611 Hansen Way, Palo Alto, California 94303, USA, 1980).
2. Varian Associates, Inc. *Digital Interface Port Operators Manual, Publication Number 87–149–635 Revision A878* (Varian Associates, Inc., 611 Hansen Way, Palo Alto, California 94303, USA, 1978).
3. SAS Institute, Inc., *SAS User's Guide* (SAS Institute, Inc., Box 8000, Cary, North Carolina 27511, USA, 1981).

**SIXTH INTERNATIONAL CONGRESS OF PESTICIDE CHEMISTRY**

*10–15 August 1986, Ottawa, Ontario, Canada*

Sponsored by IUPAC, the conference has eight main topics:

Synthesis of pesticides and bioregulators

Chemistry and bioactivity of natural products and prototypes

Chemical and biological basis of pesticide activity and resistance

Formulation chemistry and technology

Pesticide residue methodology

Environmental fate and effects

Metabolism in plants and animals

Pesticide toxicology and human health

Regulatory decision-making.

Two special symposia are also planned: Quality assurance of methods; and Biotechnology and agriculture.

*More information from Dr R. Greenhalgh, Agriculture Canada Research Centre, University Sub Post Office, London, Ontario, Canada N6A 5B7*

